# Silencing of Pepper *CaFtsH1* or *CaFtsH8* Genes Alters Normal Leaf Development

**DOI:** 10.3390/ijms24054927

**Published:** 2023-03-03

**Authors:** Kai Xu, Ning Li, Yiwen Zhang, Shenghua Gao, Yanxu Yin, Minghua Yao, Fei Wang

**Affiliations:** Hubei Key Laboratory of Vegetable Germplasm Innovation and Genetic Improvement, Institute of Industrial Crops, Hubei Academy of Agricultural Sciences, Wuhan 430070, China

**Keywords:** pepper, *CaFtsH1*, *CaFtsH8*, chloroplast development, virus-induced gene silencing

## Abstract

Filamentation temperature-sensitive H (*FtsH*) is a proteolytic enzyme that plays an important role in plant photomorphogenesis and stress resistance. However, information regarding the *FtsH* family genes in pepper is limited. In our research, through genome-wide identification, 18 members of the pepper *FtsH* family (including five *FtsHi* members) were identified and renamed based on phylogenetic analysis. *CaFtsH1* and *CaFtsH8* were found to be essential for pepper chloroplast development and photosynthesis because *FtsH5* and *FtsH2* were lost in Solanaceae diploids. We found that the *CaFtsH1* and *CaFtsH8* proteins were located in the chloroplasts and specifically expressed in pepper green tissues. Meanwhile, *CaFtsH1* and *CaFtsH8*-silenced plants created by virus-induced gene silencing exhibited albino leaf phenotypes. In addition, *CaFtsH1*-silenced plants were observed to contain very few dysplastic chloroplasts and lost the capacity for photoautotrophic growth. Transcriptome analysis revealed that the expression of chloroplast-related genes such as those coding the photosynthesis-antenna protein and structural proteins was downregulated in *CaFtsH1*-silenced plants, resulting in the inability to form normal chloroplasts. This study improves our understanding of pepper chloroplast formation and photosynthesis through the identification and functional study of *CaFtsH* genes.

## 1. Introduction

The photoreaction phase of plant photosynthesis is primarily completed on the thylakoid membrane, which contains four major complexes: PS I, ATP synthase, PS II, and cytochrome b6/f [1,2]. The biological processes of light energy absorption, transfer, and conversion in photosynthesis are all completed in PS I and PS II, both of which comprise peripheral light-harvesting complexes (LHC) and core complexes. Among these, the gene families encoding PS I and PS II core complexes are named *Psa* and *Psb*, respectively, and the gene families corresponding to LHC in PS I and PS II are named *Lhca* and *Lhcb*, respectively [3]. There are 14 LHC proteins in angiosperms, including LHCA1-6 in PS I and LHCB1-8 in PS II, whereas the PS II core complex comprises D1 (PsbA), CP43 (PsbC), D2 (PsbD), CP47 (PsbB), and other subunits [4,5].

To avoid photoinhibition, photosynthetic organisms have established various mechanisms to reduce the photodamage caused by excess light energy, and the PS II repair cycle is one of these [6,7]. This process has been studied in many species and is mostly conserved among various photosynthetic organisms. The PS II repair process can be divided into the following stages: occurrence of D1 protein photodamage in the light-induced reaction center, partial disassembly of the PS II-LHC II supercomplex to expose damaged D1, selective hydrolysis of the photodamaged D1 protein, and de novo synthesis of D1 and fabrication of a complete PS II core complex [8,9]. The whole process is extremely important for restoring and maintaining photosynthetic efficiency. In higher plants, the degradation of light-damaged D1 is carried out by FtsH and Deg proteases [10,11,12]. FtsH is an ATP-dependent protease with a transmembrane domain found in bacteria and has been studied in numerous plants, including rice [13], Arabidopsis [14], wheat [15], soybean [16], peanut [17], tomato [18], tobacco [19], pear [20], rapeseed [21], and alfalfa [22]. Most prokaryotes have only one FtsH protein, whereas multiple FtsH proteins exist in plants, where they degrade substrates in the form of heterohexameric complexes [23,24].

Among higher plants, the FtsH protein of Arabidopsis has been extensively studied. The Arabidopsis FtsH protein family comprises 17 members, including 12 FtsH proteins (FtsH1-12) and 5 FtsHi proteins (FtsHi1-5) that have lost their proteolytic function [14]. Among the 12 FtsH proteins, FtsH10, FtsH4, and FtsH3 are located in mitochondria, whereas the other nine FtsH proteins are located in the chloroplast [25]. Heterohexameric complexes were formed by four FtsH proteins (type A, FtsH1/5 proteins; type B, FtsH2/8 proteins) in chloroplasts [26]. This complex is localized in the thylakoid membrane through an N-terminal transmembrane domain, participates in the turnover of D1 proteins in the PS II repair cycle, and affects the assembly and degradation of other transmembrane subunits in the photosynthetic system [27,28]. The presence of at least one protein of each type is obligatory for chloroplastogenesis and normal photosynthesis. Consequently, mutants lacking either type A or B protease will produce a lethal phenotype [13,29,30,31]. Notably, FtsH has a high turnover rate, which is essential for light stress response [32,33]. EngA, a GTP-binding protein involved in FtsH protein turnover, is localized in chloroplasts and negatively regulates the stability of FtsH proteins through interaction with its ATPase domain [34].

The functions and molecular mechanism of pepper FtsH proteins have been sparsely investigated. In this study, we conducted genome-wide identification as well as comparative and expression pattern analyses of *FtsH* genes in pepper (*Capsicum annuum*) and found that *CaFtsH1* and *CaFtsH8* may be essential for chloroplastogenesis. To explore the underlying molecular mechanisms, we generated plants with silenced *CaFtsH1* and *CaFtsH8* and resulting albino seedling phenotypes using virus-induced gene silencing (VIGS) technology. By combining phenotypic and RNA sequencing analysis, we found that *CaFtsH1* could play a vital role in pepper chloroplast development. Our findings contribute to improving the fundamental comprehension of the Solanaceae *FtsH* family genes and laying the foundation for elucidating the function of *FtsH* genes in pepper photosynthesis.

## 2. Results

### 2.1. Identification of FtsH Proteins in Solanaceae

A total of 51 putative FtsH proteins (18 in *C. annuum*, 16 in *Solanum lycopersicum*, and 17 in *Solanum melongena*) were identified using two BLAST-based methods (BLASTp and HMMER search). These FtsH proteins were subsequently renamed based on their phylogenetic relationships and the closest Arabidopsis homologs. The physical and chemical properties of these FtsH proteins, including length, molecular weight, isoelectric point, and subcellular localization prediction, were analyzed. The lengths of the FtsH proteins ranged from 405 to 1248 amino acids, with molecular weight ranging from 44.65 to 144.07 kDa (Tables S1–S3). The isoelectric points were between 9.66 and 4.97. The results of subcellular localization prediction indicated that most FtsH proteins were located in the chloroplast or nucleus, similar to those in *Arabidopsis thaliana*.

### 2.2. Phylogenetic and Motif Analysis of FtsH Proteins

**Figure 1:**
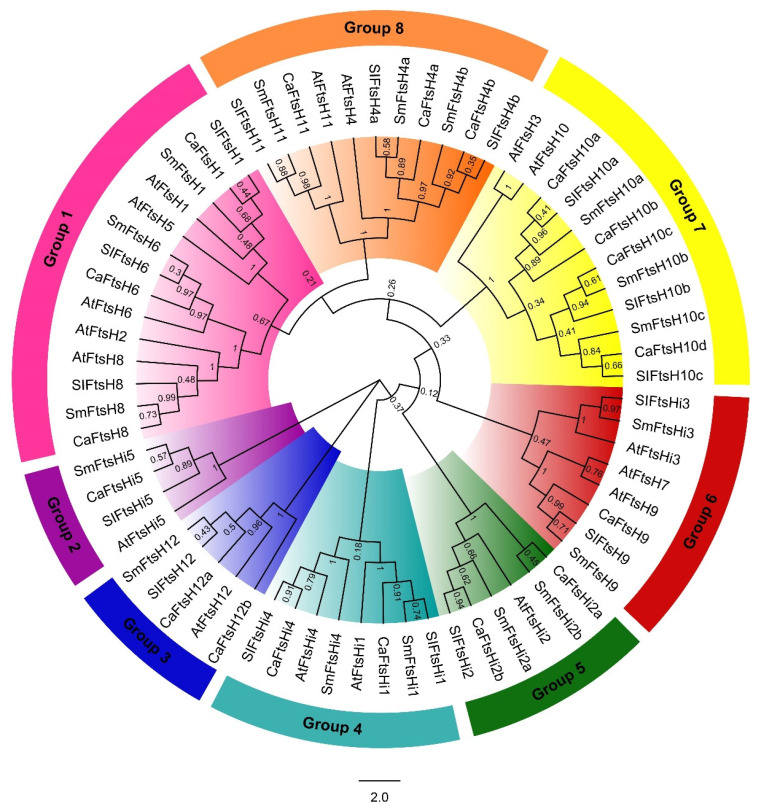
Rootless phylogenetic tree of FtsH proteins identified from pepper, tomato, eggplant, and Arabidopsis using MEGA X software v10.2 based on the maximum likelihood method. The proteins were divided into eight groups and labeled with different colors. The proteins were renumbered based on their evolutionary relationships. The number on the branches represent the bootstrap values calculated via a bootstrap test with 1000 replicates.

To elucidate the phylogenetic relationships of FtsH proteins in Solanaceae species, the amino acid sequences of FtsH proteins were identified from pepper (*C. annuum*), eggplant (*S. melongena*), tomato (*S. lycopersicum*), and Arabidopsis to draw an evolutionary tree. A total of 68 protein sequences were obtained, which were divided into groups 1 to 8 based on sequence homology (Figure 1). Group 2 was the smallest group with four members, and group 1 was the largest with fourteen members. Regarding the proteins identified in pepper, group 1 contained CaFtsH1, 6, and 8; group 3 contained CaFtsH12a and 12b; group 4 contained CaFtsHi4 and 5; group 5 contained CaFtsHi2a and 2b; group 7 contained CaFtsH10a, 10b, 10c, and 10d; and group 8 contained CaFtsH4a, 4b, and 14. Group 2 contained only CaFtsHi5 and group 6 contained CaFtsH9.

**Figure 2:**
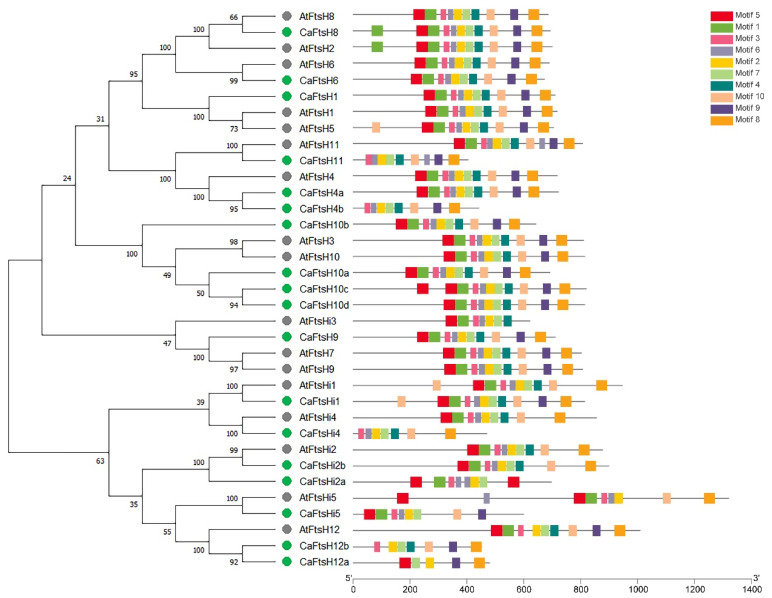
Phylogenetic relationships and conserved motif distributions of FtsH proteins in pepper and Arabidopsis. The phylogenetic tree was constructed based on the complete alignment of 35 FtsH proteins using the maximum-likelihood method. The proteins of pepper and Arabidopsis are distinguished by green and gray circles, respectively. The numbers beside the branches indicate the bootstrap support values by percentage that were obtained using 1000 iterations.

The FtsH protein sequences from Arabidopsis and pepper were adopted to draw another evolutionary tree to analyze the phylogenetic associations of the CaFtsH family proteins. Four pairs of FtsH proteins, namely, CaFtsH4a and 4b, CaFtsH10c and 10d, CaFtsH12a and 12b, and CaFtsHi2a and CaFtsHi2b, exhibited high sequence similarity (Figure 2). Compared with Arabidopsis FtsH proteins, AtFtsH6 and CaFtsH6, AtFtsH11 and CaFtsH11, AtFtsHi1 and CaFtsHi1, AtFtsHi2 and CaFtsHi2b, AtFtsHi4 and CaFtsHi4, and AtFtsHi5 and CaFtsHi5 showed the greatest homology, with bootstrap values exceeding 99%.

The structural characteristics of pepper FtsH proteins were studied using the MEME website and TBtools software v1.100, resulting in the identification of 10 conserved motifs (Figure 2). The number of FtsH motifs ranged from 5 (CaFtsH12a) to 11 (CaFtsH8). Motifs 2, 3, 6, and 7 were commonly distributed in most FtsH proteins, except for CaFtsH12a and CaFtsH12b. Multiple proteins had a complete 10-motif conserved structure, including CaFtsH1, 4a, 6, 8, 9, 10a, 10b, 10c, 10d, and CaFtsHi1. Proteins in the same clade generally contained similar arrangements and numbers of conserved motifs, indicating homologous functions among these CaFtsH members.

### 2.3. Essential Roles of CaFtsH1 and CaFtsH8 in Pepper

**Figure 3:**
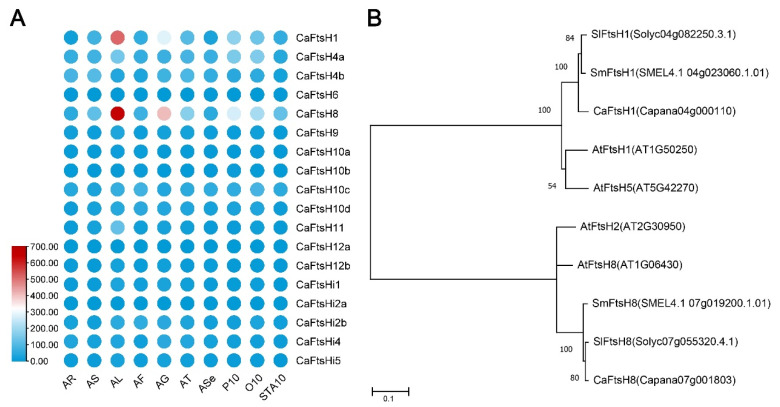
Expression pattern and phylogenetic analysis of *CaFtsHs*. (**A**) Heat map of the expression levels of 18 *CaFtsH* genes in diverse pepper tissues utilizing transcriptome analysis, including roots (AR), stems (AS), leaves (AL), flowers (AF), pulps (AG), placentas (AT), seeds (ASe), petals (P10), ovaries (O10), and stamens (STA10). (**B**) Phylogenetic analysis of AtFtsH1, 2, 5, 8, and their six closest homologs in *C. annuum*, *S. lycopersicum*, and *S. melongena*. The branch lengths represent the evolutionary distance. Numbers next to the branches denote the bootstrap values with 1000 replicates (bootstrap test).

To examine the expression characteristics of *CaFtsH* genes in diverse tissues, the transcriptome data of pepper roots, stems, leaves, flowers, pulps, placentas, seeds, petals, ovaries, and stamens were downloaded from the PepperHub Database. The heat map showed that, except for *CaFtsH1* (*Capana04g000110*) and *CaFtsH8 (Capana07g001803)*, the *CaFtsH* genes did not exhibit significant tissue-specific expression. Notably, the expression levels of *CaFtsH1* and *CaFtsH8* were higher in the leaves and pulp than in other organs (Figure 3A). Through conserved domain analysis of pepper FtsH proteins, we found that the domains of CaFtsH1 and CaFtsH8 were conserved and had the complete structure and function of FtsH proteases (Figure S1).

Functional and evolutionary analyses of homologous genes were performed to clarify the reasons for the high expression of *CaFtsH1* and *CaFtsH8* in pepper leaves. In Arabidopsis, heterohexameric complexes comprised four FtsH proteins (type A, FtsH1/5 proteins; type B, FtsH2/8 proteins) in the chloroplasts [26]. Mutants lacking either type A or type B proteases have been reported to produce lethal phenotypes in multiple species [13,29,30,31]. Notably, based on phylogenetic analysis, three species of Solanaceae (pepper, tomato, and eggplant) were found to lack homologous proteins for AtFtsH2 and AtFtsH5 (Figure 3B). These three species contained only one homolog of FtsH1 (CaFtsH1, SlFtsH1, and SmFtsH1) and one homolog of FtsH8 (CaFtsH8, SlFtsH8, and SmFtsH8). These findings indicate that FtsH1 and FtsH8 proteins may be necessary for the PS II repair cycle, photosynthesis, and chloroplast development in Solanaceae, which would explain the high expression levels of *CaFtsH1* and *CaFtsH8* in pepper leaves.

### 2.4. Expression Pattern and Subcellular Location of CaFtsH1 and CaFtsH8

**Figure 4:**
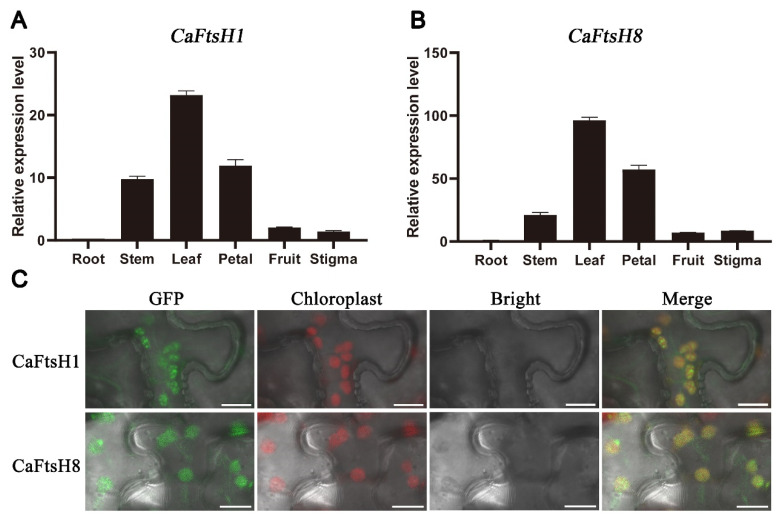
Expression patterns and subcellular localization of CaFtsH1 and CaFtsH8. (**A**,**B**) Expression patterns of *CaFtsH1* and *CaFtsH8* in the roots, leaves, petals, stems, fruits, and stigmas of the *C. annuum* cultivar Z101, as determined based on RT-qPCR utilizing the expression of *CaUBI-3* as a reference. Values are expressed as average ± SD (*n* = 3). (**C**) Subcellular localization of CaFtsH1 and CaFtsH8 in *N. benthamiana* leaves. Merged pictures contain the green fluorescence of CaFtsH1 or CaFtsH8 (first panels), chloroplast red fluorescence (second panels), and the bright-field image (third panels). Bars = 12 μm.

To better characterize the functions of *CaFtsH1* and *CaFtsH8* in pepper, RT-qPCR was applied to evaluate their expression patterns in different tissues (roots, stems, leaves, petals, fruits, and stigmas). As shown in the histograms, *CaFtsH1* and *CaFtsH8* exhibited extremely high expression in leaves, followed by petals and stems, whereas in the roots, the expression levels of the two genes were negligible (Figure 4A,B). In conclusion, *CaFtsH1* and *CaFtsH8* were found to be mainly expressed in green tissues, which was generally consistent with the results of the transcriptome data and subcellular localization prediction.

To verify the subcellular localization of CaFtsH1 and CaFtsH8, two fusion vectors (pMDC83-GFP-CaFtsH1 and pMDC83-GFP-CaFtsH8) were constructed and transiently expressed in *Nicotiana benthamiana* leaves. As expected, the green fluorescent signals of CaFtsH1 and CaFtsH8 closely overlapped with chloroplast auto-fluorescence, illustrating that CaFtsH1 and CaFtsH8 were both localized in the chloroplasts (Figure 4C).

### 2.5. Gene Silencing of CaFtsH1 or CaFtsH8 Confers Albino Phenotypes

**Figure 5:**
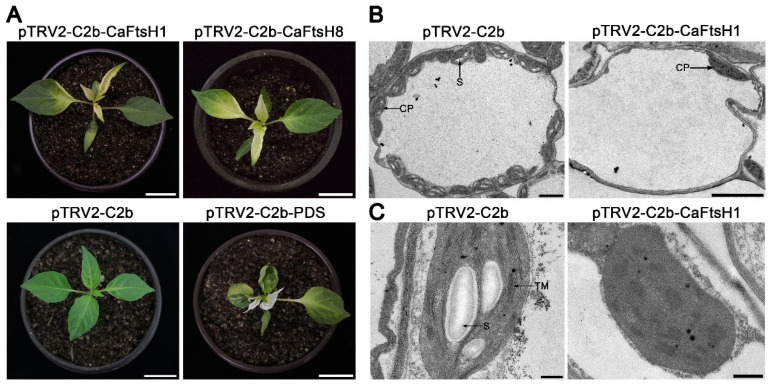
Phenotype and chloroplast ultramicrostructure observation of gene silenced plants via the VIGS system. (**A**) Phenotypes of representative individuals of pTRV2-C2b-CaFtsH1 and pTRV2-C2b-CaFtsH8-positive plants, and their positive (pTRV2-C2b-PDS) and negative (pTRV2-C2b) controls. (**B**,**C**) Transmission electron micrographs of chloroplasts in negative control and *CaFtsH1-*silenced plants. S-starch grains, CP-chloroplast, and TM-thylakoid membrane. Bars: (**A**) 3 cm; (**B**) 3 µm; (**C**) 500 nm.

To further identify the functions of *CaFtsH1* and *CaFtsH8*, two TRV-based VIGS vectors (pTRV2-C2b-CaFtsH1 and pTRV2-C2b-CaFtsH8) carrying two specific 200–300 bp fragments of the coding sequence were constructed. The two plasmids were integrated through *Agrobacterium*-mediated injection into the underside of pepper cotyledons. For positive and negative controls, we used pTRV2-C2b-PDS (Phytoene desaturase) and pTRV2-C2b, respectively. Pepper plants inoculated with pTRV2-C2b-PDS showed photobleaching phenotypes, whereas pTRV2-C2b (the empty vector) exhibited completely normal leaf phenotypes (Figure 5A). Meanwhile, positive pTRV2-C2b-CaFtsH1 and pTRV2-C2b-CaFtsH8 plants exhibited albino leaf phenotypes (Figure 5A). The two recombinant vectors (pTRV2-C2b-CaFtsH1 and pTRV2-C2b-CaFtsH8) were injected into 150 individuals via three separate injections, achieving silencing efficiencies of 35.8% and 39.7%, respectively. Based on RT-qPCR, the expression levels of *CaFtsH1* and *CaFtsH8* was significantly reduced in the respective silenced plants, illustrating the accuracy of VIGS results (Figure S2).

Owing to the formation of the same heterohexamer complex involved in the PS II repair cycle, mutants lacking type A (FtsH1/5) or type B (FtsH2/8) proteases produced albino leaf phenotypes, abnormal chloroplast structures and affected the expression of photosynthesis-related genes [13,21,31]. Considering that CaFtsH1 and CaFtsH8 proteins have similar functions, only *CaFtsH1*-silenced plants were selected for follow-up research. Consequently, the ultramicrostructure of negative control and pTRV2-C2b-CaFtsH1-silenced plants chloroplasts were observed by transmission electron microscopy (TEM). As illustrated in Figure 5, plump chloroplast structures, starch grains, and stacked thylakoid membranes were discovered in negative controls, whereas very few dysplastic chloroplasts and the absence of starch grains were observed in the pTRV2-C2b-CaFtsH1-silenced plants (Figure 5B,C), demonstrating that the development of chloroplasts was severely impaired in the silenced plants. These results indicate that *CaFtsH1* might be essential for chloroplast development and photosynthesis in pepper.

### 2.6. Analysis of Differentially Expressed Genes in CaFtsH1-Silenced Plants

To understand the molecular function of *CaFtsH1* in the development of pepper chloroplasts, the second leaves from the top of *CaFtsH1*-silenced plants and negative controls were used for transcriptome analysis. A total of 197,480,520 clean reads were obtained from the six samples after eliminating low-quality reads, and more than 86% of the reads was uniquely mapped to the zunla-1 genome [35] (Table S4). Based on Pearson’s correlation analysis, the correlation between biological replicates was found to be over 96%, and the RNA-seq data were deemed reliable (Figure S3). Subsequently, the expression levels of pepper genes were estimated, and a total of 5522 differentially expressed genes (DEGs) were identified. In *CaFtsH1-*silenced plants, there were 2920 upregulated and 2602 downregulated genes, respectively.

**Figure 6:**
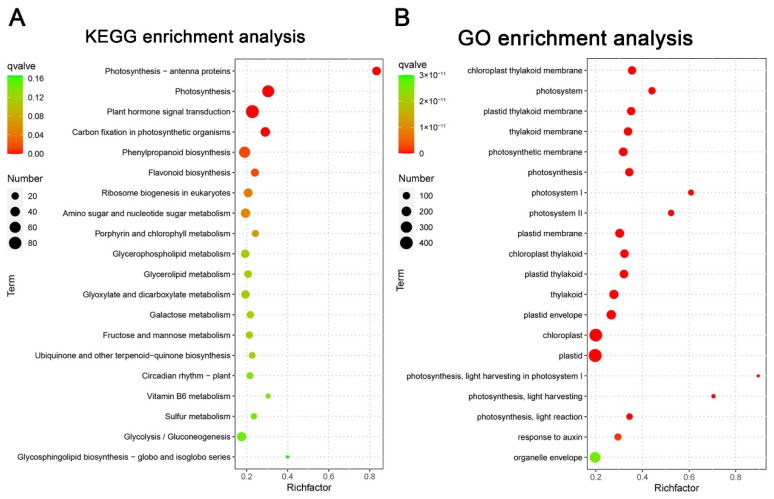
RNA-seq analysis of DEGs in *CaFtsH1*-silenced plants compared with those in the negative controls. (**A**) KEGG pathway enrichment analysis of DEGs. (**B**) GO enrichment analysis of DEGs.

The Kyoto Encyclopedia of Genes and Genomes (KEGG) pathway analysis was conducted to determine the effect of *CaFtsH1* on the biological pathways of pepper. Accordingly, 120 pathways were found to be disturbed in *CaFtsH1-*silenced plants. As shown in Figure 6A, the 20 most significantly affected pathways were identified, which included pathways associated with photosynthesis-antenna proteins, photosynthesis, plant hormone signal transduction, and carbon fixation in photosynthetic organisms. The DEGs were then annotated according to the Gene Ontology (GO) database and divided into three groups, including cellular component (CC), molecular function (MF), and biological process (BP). The 20 most significant GO terms are shown in Figure 6B, none of which belonged to the MF category. Only terms belonging to the CC and BP groups, including plastid thylakoid, thylakoid, plastid envelope, chloroplast, plastid, and photosynthesis were enriched, demonstrating that these terms were influenced by *CaFtsH1*.

### 2.7. Suppression of Photosynthesis-Related Genes in CaFtsH1-Silenced Plants

**Figure 7:**
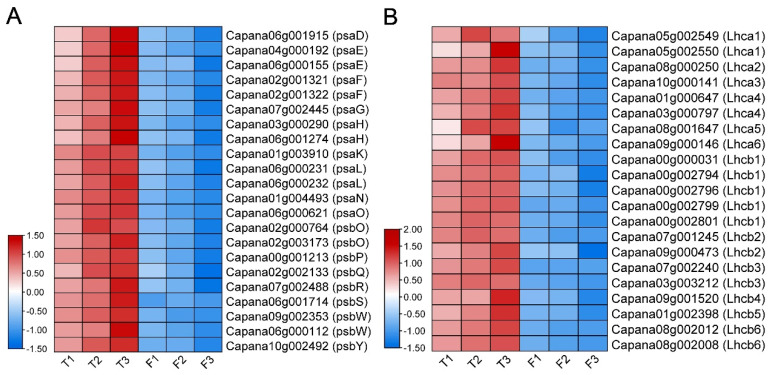
Heat maps of photosynthesis-related genes in PS I and PS II. (**A**) Heat map of 22 structural protein genes (13 *Psa* subunit genes and 9 *Psb* subunit genes). (**B**) Heat map of 21 antenna protein genes (8 *Lhca* genes and 13 *Lhcb* genes). T1−T3 and F1−F3 represent three biological replicates of negative controls (pTRV2-C2b) and *CaFtsH1*-silenced plants (pTRV2-C2b-CaFtsH1), respectively.

FtsH participates in the PS II repair cycle of Arabidopsis as a major enzyme. According to the TEM and KEGG analysis results, the photosynthetic system of *CaFtsH1*-silenced plants was disturbed, as indicated by the altered expression of the photosynthesis-antenna proteins and structural proteins. Through VIGS, *CaFtsH1* expression was notably reduced in the silenced plants (Table S5). Meanwhile, the expression levels of 22 structural protein genes (13 *Psa* genes and 9 *Psb* genes) (Figure 7A) and 21 antenna protein genes (8 *Lhca* genes and 13 *Lhcb* genes) (Figure 7B) were decreased in *CaFtsH1*-silenced plants. The suppression of other pathways, such as carbon fixation in photosynthetic organisms, ribosome biogenesis in eukaryotes, and plant hormone signal transduction, may have been caused by abnormal chloroplast development and cessation of photosynthesis in the silenced plants. The accuracy of RNA-seq results was validated through RT-qPCR detection of 20 genes (Figure S4A,B, Table S5). Our findings suggest that the silencing of *CaFtsH1* expression inhibited the expression of photosynthesis-related genes, thereby affecting the morphological establishment of PS I and PS II and the normal operation of the photosynthetic system.

### 2.8. Silencing of CaFtsH1 Expression Influences CaPsbA Expression

In Arabidopsis, FstH is involved in the degradation of damaged D1 (PsbA), while EngA negatively regulates the stability of FtsH. Through sequence alignment, the homolog of chloroplast *PsbA* gene (NCBI Gene ID: 13540179) in pepper was identified. We also found that the expression of *CaPsbA* was lower in *CaFtsH1*-silenced plants (Figure S4C). Similarly, only one *EngA* homolog gene (*Capana11g000842*) was identified in pepper; however, the expression of *CaEngA* in both negative controls and *CaFtsH1*-silenced plants was very low, and no significant difference was observed (Table S5). Using yeast two-hybrid pairwise assays, it was further determined that there was no interaction between CaFtsH1 and CaEngA (Figure S5). Briefly, silencing of *CaFtsH1* decreased the expression of *CaPsbA*, indicating that the PS II repair cycle may have been affected.

## 3. Discussion

*FtsH* is a conserved gene family that is widespread in all higher plants and essential for early chloroplast development and photosynthesis. In addition, the *FtsH* family genes contribute to the tolerance of stress factors such as high temperature, drought, and high salinity [19,36]. Recently, genome-wide analysis has been intensively used to study *FtsH* in many species, but not in pepper. In this study, 18 members of the pepper FtsH family (including five FtsHi members) were identified and renamed based on their phylogenetic relationships. Among these, *CaFtsH1* and *CaFtsH8* were found to be highly expressed in pepper leaves and essential for pepper leaf development and photosynthesis.

The heterohexameric complex, composed of FtsH1, 5, 2, 8, is essential for photosynthesis and normal growth and development in higher plants. Mutants lacking either type A (FtsH1/5) or type B (FtsH2/8) proteases have been reported to produce lethal phenotypes in multiple species [13,29,30,31]. Through phylogenetic analysis of FtsH proteins in Arabidopsis, pepper, tomato, and eggplant, FtsH5 and FtsH2 were found to be lost in Solanaceae diploids. We hypothesize that the last remaining FtsH1 and FtsH8 genes may be crucial for the survival of Solanaceae. This inference was verified in peppers by VIGS, TEM observation, and transcriptome analysis. This indicates that *FtsH1* and *FtsH8* may be extremely important for chloroplast formation, normal photosynthesis, and survival of tomato and eggplant.

In plants, the FtsH complex participates in the rapid degradation of damaged D1 during the PS II repair cycle. Subcellular localization showed that CaFtsH1 and CaFtsH8 proteins were located in chloroplasts. Therefore, *CaFtsH1* and *CaFtsH8* probably encode the FtsH1 and FtsH8 proteins, respectively, and are potentially involved in the formation of heterohexamer complexes and the hydrolysis of light-damaged D1. In silenced plants, the lack of FtsH1 or FtsH8 proteins prevented the synthesis of these complexes as well as the normal operation of photosynthetic systems, resulting in the albino leaf phenotypes of pepper. In this study, *CaPsbA* (D1 coding gene) expression was lower in *CaFtsH1*-silenced plants, suggesting that the PS II repair cycle may have been affected. In contrast to previous reports, the results of this study indicate that *CaEngA* may not be involved in the regulation of the FtsH1 protein in pepper.

Alleviating photoinhibition is an important means to improve photosynthetic efficiency. Although the details of the PS II repair cycle are still being explored, its broad application potential for alleviating photoinhibition and improving photosynthetic efficiency is generally recognized. In a previous study, the photosynthetic efficiency of *A. thaliana* and *N. benthamiana* as well as the yield of rice were significantly increased by overexpressing and targeting D1 to chloroplasts [37]. Meanwhile, through determining the biomass of transgenic T_1_ generation plants, it was found that the increase of *FtsH1* copy number was beneficial to the biomass accumulation of *Brassica napus* [31]. It has been speculated that the photosynthetic efficiency of pepper could be improved by regulating the expression of *FtsH* family genes, thereby increasing the accumulation of pepper biomass.

## 4. Materials and Methods

### 4.1. Identification of FtsH Proteins in Solanaceae

To identify members of the FtsH family in pepper, tomato, and eggplant, multiple databases and methods were utilized. First, the *A. thaliana* FtsH family protein sequences were acquired from the TAIR online database [38], and adopted as the query to perform local BLASTp searches in the Solanaceae Genomics Network (https://solgenomics.net/; accessed on 7 December 2022) [39]. Second, the conserved FtsH domain (PF06480) sequences were downloaded from the Pfam database (https://www.ebi.ac.uk/interpro/; accessed on 7 December 2022) [40] to perform a HMMER search. Third, the final identification of FtsH family members and protein sequences was achieved by combining the results of BLASTp and HMMER. The conservative domains of candidate FtsH proteins was then manually examined using the NCBI Batch CD-Search Tool.

### 4.2. Properties, Structure, and Conserved Motifs of FtsH Proteins

The physicochemical properties of FtsH proteins, such as molecular weights and isoelectric points, were evaluated by utilizing the ExPASy database (https://web.expasy.org/compute_pi/; accessed on 7 December 2022) [41]. Prediction of subcellular localization was accomplished by utilizing the Plant-mPloc website (http://www.csbio.sjtu.edu.cn/bioinf/plant-multi/; accessed on 7 December 2022) [42]. MEME version 5.5.0 (https://meme-suite.org/meme/tools/meme; accessed on 7 December 2022) [43] was used to identify conserved motifs in FtsH protein sequences, with the specific parameters: any number of repetitions was selected as the site distribution, and up to 10 motifs were retrieved. The patterns of conserved motifs were then visualized by TBtools v1.100 [44]. Analysis of protein structure and conserved domains were conducted using Jalview version 2.11.2.5 [45].

### 4.3. Phylogenetic Analysis of FtsH Proteins

Phylogenetic analysis was performed using full-length protein sequences of the plants (*A. thaliana*, *C. annuum*, *S. lycopersicum*, and *S. melongena*) downloaded from the Solanaceae Genomics Network. Phylogenetic trees were generated using MEGA X software v10.2 [46]. The amino acid sequences of FtsH proteins were aligned using MUSCLE, and the maximum likelihood method and most suitable model (LG + G + I) were adopted to infer evolutionary history. Bootstrap was calculated with 1000 replicates to ensure accuracy. The final phylogenetic tree was viewed and polished using FigTree V1.4.4 software [47].

### 4.4. Expression Pattern and RT-qPCR Analysis

For expression pattern analysis, transcriptome data from 10 pepper tissues (roots, stems, leaves, flowers, pulps, placentas, seeds, petals, ovaries, and stamens) were downloaded from the PepperHub Database (http://122.205.95.132/index.php; accessed on 7 December 2022) [48]. The expression patterns of each *FtsH* gene were visualized using heat maps generated in TBtools v1.100 software [44]. Meanwhile, the expression patterns of *CaFtsH1* and *CaFtsH8* were verified using RT-qPCR. Six tissues (roots, petals, stems, tender leaves, stigmas and green fruits) of Z101 were sampled for RNA extraction using TRIzol reagent. Three biological replicates were adopted for each tissue type.

First-strand cDNA was obtained using the HiScript II 1st Strand cDNA Synthesis Kit (+ gDNA wiper) (Vazyme, Nanjing, China) according to reagent instructions. RT-qPCR reactions were carried out using three biological and three technical replicates, using the ChamQ Universal SYBR^®^ qPCR Master Mix (Vazyme) in a CFX384 Real-Time PCR Detection System (Bio-Rad, Hercules, CA, USA). The relative gene expression levels were evaluated using the 2^−∆∆Ct^ method; meanwhile, the *CaUBI-3* gene [49] was adopted as an internal PCR control. All gene-specific primers utilized in this experiment are summarized in Table S6.

### 4.5. Subcellular Localization of CaFtsH1 and CaFtsH8

The coding sequences of *CaFtsH1* and *CaFtsH8* without the stop codon were amplified and connected to the *Kpn*I/*Bam*HI sites of the pMDC83-GFP vector [50], using a ClonExpress II One Step Cloning Kit (Vazyme). The recombinant plasmids were then introduced into *Agrobacterium* strain GV3101 by utilizing the freeze–thaw strategy, and subcellular localization was observed as previously reported [51]. Three-week-old *N. benthamiana* leaves were injected with the GV3101 suspension harboring targeting vectors and then incubated at 25 °C for 3 days. GFP fluorescence signals were detected and imaged using a confocal microscope (Leica SP8; Leica, Wetzlar, Germany) at a wavelength of 488 nm.

### 4.6. Gene Function Verification of CaFtsHs by VIGS

To identify the functions of *CaFtsH1* and *CaFtsH8*, TRV-based VIGS assay was applied following a previously described method [52]. Two specific 200–300 bp coding sequence fragments were cloned and connected to the pTRV2-C2b vector using single-enzyme digestion (*Sma* I). The sequences of the two vectors (pTRV2-C2b-CaFtsH1 and pTRV2-C2b-CaFtsH8) were confirmed by sequencing and then introduced into GV3101. *Agrobacterium* was inoculated and cultured using special Luria–Bertani medium, containing 50 mg/L kanamycin, 10 mg/mL rifampin, 40 μM acetosyringon, and 10 µM 2-Morpholinoethanesulphonic acid. After incubation at 28 °C (220 rpm/min) for 16 h, *Agrobacterium* cells were collected (5000 rpm, 10 min) and resuspended in the infiltration buffer (200 mM MgCl_2_, 150 µM acetosyringon). The concentration of the resuspended solution was then adjusted to OD_600_ = 0.1 and placed in a dark environment for 2–3 h before use.

For subsequent injection steps, the above suspensions containing pTRV1 and pTRV2-C2b or its derivatives (pTRV2-C2b-PDS, pTRV2-C2b-CaFtsH1, and pTRV2-C2b-CaFtsH8) were mixed at a 1:1 ratio and adjusted to OD_600_ = 0.004 for inoculation. Subsequently, the bacterial solutions were injected into the underside of the pepper cotyledons using 1 mL disposable syringes without needles. At the time of injection, the peppers were in the cotyledon stage, the two cotyledons had just fully expanded, and there were no true leaves. pTRV2-C2b-PDS and pTRV2-C2b were adopted as positive and negative controls for VIGS, respectively. After injection, the pepper plants were cultured in the dark at 16 °C for 56 h and then transferred to a normal greenhouse environment (16 h light/8 h dark cycles, 25 °C) for phenotypic observation.

### 4.7. TEM Analysis

VIGS-positive plants of the vector pTRV2-C2b-CaFtsH1 and their negative controls were used for TEM analysis. The experiment was conducted as previously described [53]. The fresh leaves of the peppers were cut into 1–2 mm slices and fixed in a solution of 2.5% glutaraldehyde at 4 °C. After osmic acid fixation, gradient ethanol dehydration, infiltration, and embedding, the tissue samples were sliced into 80–100 nm thick sections using an ultramicrotome (Leica UC7; Leica, Wetzlar, Germany) and then observed under a Tecnai G2 20 TWIN transmission electron microscope (FEI Company, Hillsboro, OR, USA).

### 4.8. Acquisition and Analysis of Transcriptome Data

The total RNA of pTRV2-C2b-CaFtsH1-positive plants and their negative controls was extracted for transcriptome analysis from their top second leaves at the five-leaf stage. Each type was set up with three biological replicates, and each sample contained more than 1 µg RNA for library construction. The fragment analyzer and Nanodrop 2000 (Thermo Fisher Scientific, Waltham, MA, USA) were used to ensure the concentration and quality of the RNA samples. Six libraries were constructed to produce paired-end reads using an MGISEQ-2000 sequencing platform (BGI, Shenzhen, China). Clean reads were acquired by filtering low-quality reads from raw reads using the SOAPnuke software v2.1.0 [54], with the following parameters: nRate = 0.005, lowQual = 20, and qualRate = 0.5. The quality controlled clean reads were subsequently mapped to the Zunla-1 genome using HISAT2 and Bowtie 2 [55,56]. The relative expression of all pepper genes was estimated by calculating the FPKM value using RSEM software v1.3.2 [57]. DEGs were assigned using DESeq2 [58], and the screening criteria were |Log_2_(FoldChange)| > 1.0 and FDR < 0.05. KEGG pathway enrichment and GO enrichment analyses of DEGs were completed utilizing KOBAS 3.0 and TBtools v1.100 [44,59]. TBtools was also used to draw gene expression heat maps.

### 4.9. Yeast Two-Hybrid Pairwise Assays

Gal4-based yeast-two-hybrid analyses were performed using the Matchmaker™ Library Construction & Screening Kits (Clontech, San Francisco, CA, USA) following the manufacturer’s protocol. Three solid media were used in the experiment: SD/-Trp/-Leu/X-α-Gal (DDO/X), SD/-Trp/-Leu/-His/X-α-Gal (TDO/X), and SD/-Trp/-Leu/-His/-Ade/X-α-Gal/AbA (QDO/X/A). The complete coding sequences of *CaFtsH1* (*Capana04g000110*) and *CaEngA* (*Capana11g000842*) were amplified and connected into the vectors pGBKT7 and pGADT7, respectively. For the evaluation of protein–protein interactions, the recombinant pGADT7-CaFtsH1 and pGBKT7-CaEngA constructs were co-transformed into AH109 yeast cells. Subsequently, the co-transformants were coated onto the DDO/X or QDO/X/A plates, and the results were observed after 3–5 days of incubation at 30 °C. Experiment results indicate that the two vectors (pGADT7-CaFtsH1 and pGBKT7-CaEngA) did not show self-activation with empty vector controls. The construct primers are listed in Table S6.

## Data Availability

All data generated or analyzed during this study are included in this published article and its supplementary information files.

## Supplementary Materials

The following supporting information can be downloaded at: https://www.mdpi.com/article/10.3390/ijms24054927/s1.
