# Supplementary material for: Silencing of Pepper CaFtsH1 or CaFtsH8 Genes Alters Normal Leaf Development
Source: Int J Mol Sci. 2023 Mar 3;24(5):4927. doi: 10.3390/ijms24054927 (PMC10003178; doi:10.3390/ijms24054927)
Supplement: Supplementary file 1 [file ijms-24-04927-s001.zip › ijms-2261936-supplementary.pdf]

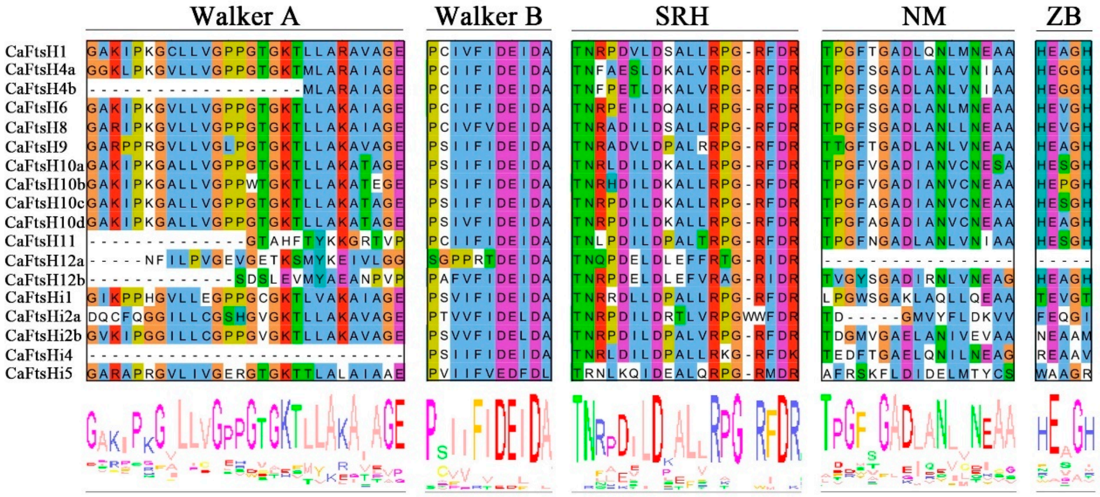

Figure S1. Conservative Domain Comparison Analysis of the *CaFtsHs* in Pepper. Walker A and Walker B were conserved motifs responsible for ATP binding and protein hydrolysis; SRH: the second region of homology, also named the AAA module; NM: non-named region of homology; ZB: Zn binding, generally a conserved HEXXH motif for Zn^{2+} binding.

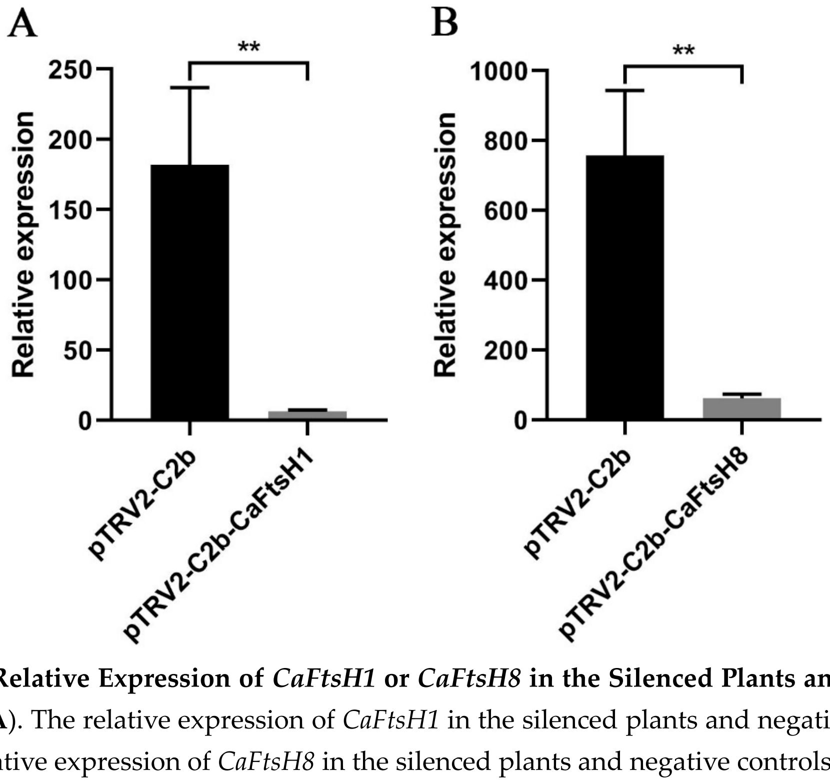

Figure S2. Relative Expression of *CaFtsH1* or *CaFtsH8* in the Silenced Plants and Negative Controls. (**A**). The relative expression of *CaFtsH1* in the silenced plants and negative controls. (**B**). The relative expression of *CaFtsH8* in the silenced plants and negative controls. Values are expressed as average ± SD (n = 3). The significant differences were identified using a Student's t-test, asterisks indicate a significant difference: **P < 0.01.

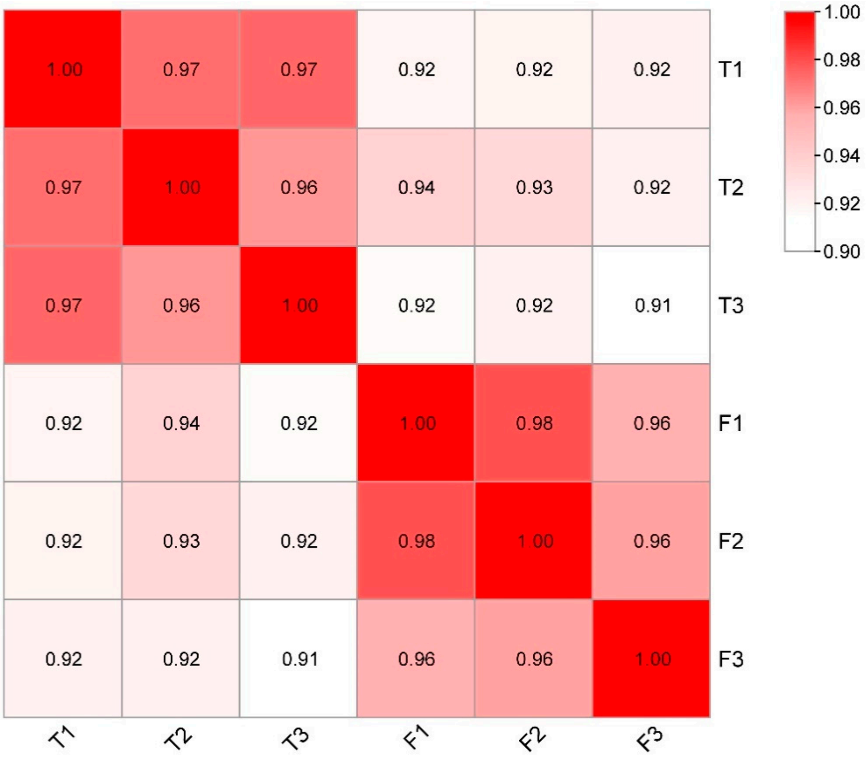

Figure S3. Correlation Analysis of Transcriptome Data Using Pearson Correlation Coefficients. Among them, T1-T3 and F1-F3 represent three biological replicates of negative control (pTRV2-C2b) and silenced plants (pTRV2-C2b-CaFtsH1), respectively.

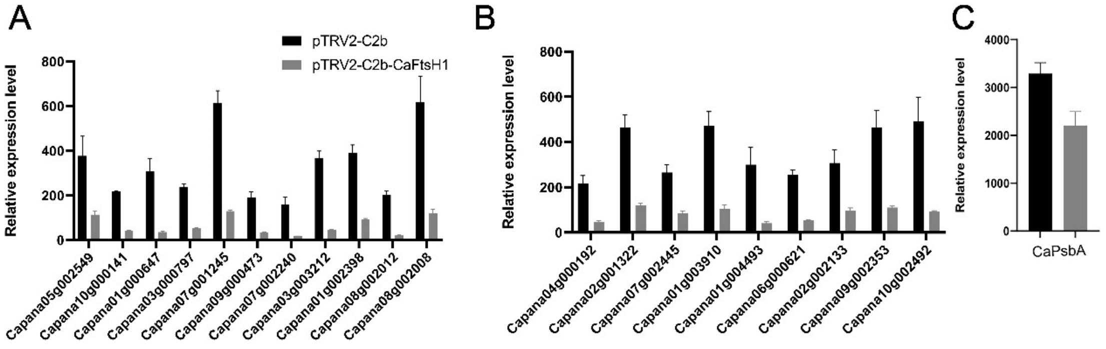

Figure S4. RT-qPCR Validation of DEGs in Transcriptome Data. (**A**). RT-qPCR confirmation of 11 antenna protein genes. (**B**). RT-qPCR confirmation of 9 structural protein genes. (**C**). RT-qPCR analysis of *CaPsbA* gene. Error bars mean SDs from three biological replicates.

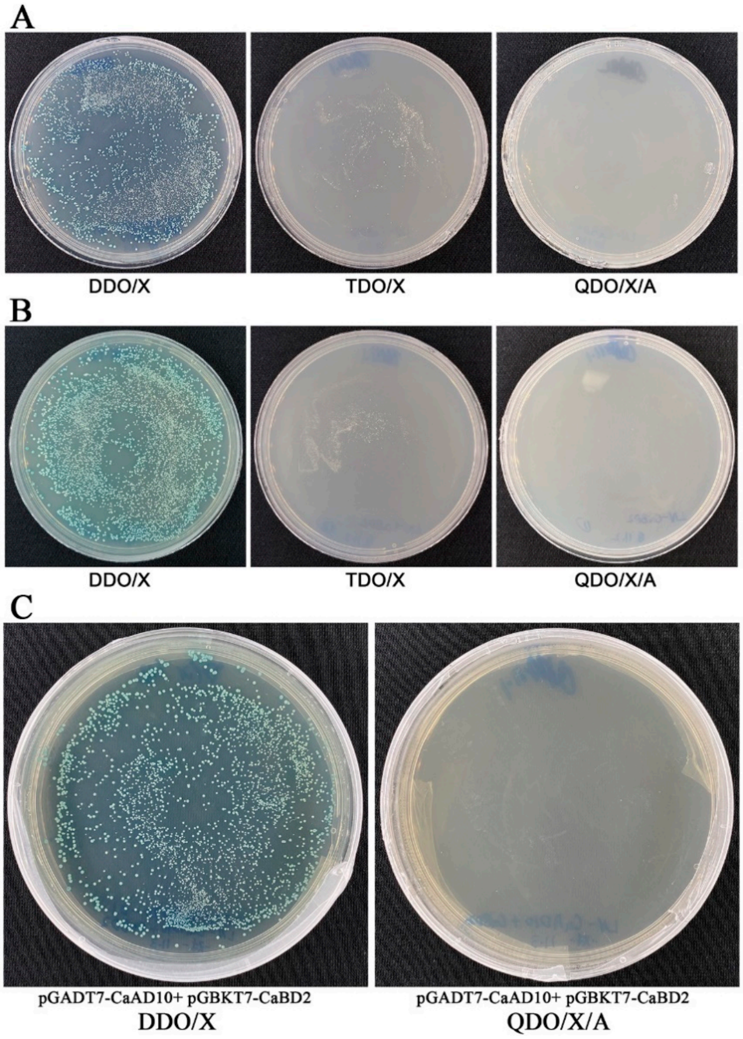

Figure S5. Point to Point Yeast Two-Hybrid Pairwise Assay of CaFtsH1 and CaEngA. (**A**). Detection of pGADT7-CaFtsH1 self-activation (growth of pGADT7-CaFtsH1+pGBKT7). (**B**). Detection of pGBKT7-CaEngA self-activation (growth of pGBKT7-CaEngA+pGADT7). (**C**). Yeast two-hybrid pairwise assay of pGADT7-CaFtsH1 and pGBKT7-CaEngA.

Table S1. Identification and Analysis of Putative *FtsH* Genes in *Capsicum annuum*.

Gene name	Gene ID	Start	Stop	Protein Length (aa)	pI	Molecular weight	Localization
CaFtsH1	*Capana04g000110*	1231018	1234121	710	6.00	76295.97	Chloroplast
CaFtsH4a	*Capana05g001818*	175991673	176000127	721	9.21	77708.74	Chloroplast, Nucleus
CaFtsH4b	*Capana08g000315*	36264744	36266625	442	6.49	47310.24	Chloroplast, Nucleus
CaFtsH6	*Capana02g002314**	143114499	143116982	673	5.74	72694.21	Chloroplast
CaFtsH8	*Capana07g001803**	203993885	203996616	693	6.07	74088.81	Chloroplast
CaFtsH9	*Capana00g003035*	518259225	518268144	711	5.97	76771.71	Chloroplast, Nucleus
CaFtsH10a	*Capana00g002383*	472844983	472848508	692	7.63	77093.66	Chloroplast, Nucleus
CaFtsH10b	*Capana10g001411*	152993495	152997066	643	5.68	71826.65	Chloroplast, Nucleus
CaFtsH10c	*Capana09g001789*	205277432	205286477	820	8.39	90475.26	Chloroplast, Nucleus
CaFtsH10d	*Capana10g001412*	153031473	153053529	814	7.98	89492.87	Chloroplast, Nucleus
CaFtsH11	*Capana03g002053*	43419943	43428682	405	6.29	44652.04	Chloroplast
CaFtsH12a	*Capana06g001351*	29476054	29478009	480	5.40	55362.27	Chloroplast, Nucleus
CaFtsH12b	*Capana03g003013*	29476054	29478009	452	4.97	51015.79	Chloroplast
CaFtsHi1	*Capana01g000121*	1798637	1805615	814	6.28	91365.35	Chloroplast, Nucleus
CaFtsHi2a	*Capana01g000437**	7854731	7863061	697	9.23	79854.20	Chloroplast, Nucleus
CaFtsHi2b	*Capana12g001231*	62781166	62797305	899	9.00	102400.06	Nucleus
CaFtsHi4	*Capana00g004440*	643849728	643857125	470	5.24	52378.01	Chloroplast
CaFtsHi5	*Capana02g000283*	38193733	38213616	599	8.29	67776.15	Chloroplast, Nucleus

* indicate that the gene sequences were corrected.

Table S2. Identification and Analysis of Putative *FtsH* Genes in *Solanum lycopersicum*.

Gene name	Gene ID	Start	Stop	Protein Length (aa)	pI	Molecular weight	Localization
SlFtsH1	*Solyc04g082250.3.1*	63951306	63955123	708	6.13	75980.6	Chloroplast
SlFtsH4a	*Solyc11g069950.2.1*	52607480	52616886	714	9.24	77248.26	Nucleus
SlFtsH4b	*Solyc08g063050.3.1*	50695490	50703203	716	8.86	77273.5	Chloroplast, Nucleus
SlFtsH6	*Solyc02g081550.3.1*	43453730	43456434	672	6.76	73171.09	Chloroplast
SlFtsH8	*Solyc07g055320.4.1*	63307593	63311739	705	5.95	75947.97	Chloroplast
SlFtsH9	*Solyc06g005950.3.1*	942142	951834	842	8.31	92209.47	Chloroplast, Nucleus
SlFtsH10a	*Solyc10g086540.3.1*	64476807	64482370	700	7.23	78403.82	Nucleus
SlFtsH10b	*Solyc09g010950.3.1*	4342979	4351220	813	8.71	89843.79	Chloroplast, Nucleus
SlFtsH10c	*Solyc10g086550.2.1*	64484030	64495789	813	7.93	89643.13	Chloroplast
SlFtsH11	*Solyc03g007330.3.1*	1921224	1932975	812	5.9	89145.81	Chloroplast, Nucleus
SlFtsH12	*Solyc02g079000.4.1*	41591999	41607707	1035	6.74	118406.9	Nucleus
SlFtsHi1	*Solyc08g082530.4.1*	63434117	63441792	956	8.71	107647	Chloroplast, Nucleus
SlFtsHi2	*Solyc12g044400.3.1*	58958982	58971269	860	9.44	97683.4	Nucleus
SlFtsHi3	*Solyc03g046340.3.1*	11498188	11506254	656	9.66	72773.83	Chloroplast, Nucleus
SlFtsHi4	*Solyc04g063360.3.1*	54814530	54830737	844	6.26	94947.49	Chloroplast, Nucleus
SlFtsHi5	*Solyc02g032960.2.1*	28131316	28152528	1082	9.22	125282.4	Nucleus

Table S3. Identification and Analysis of Putative *FtsH* Genes in *Solanum melongena*.

Gene name	Gene ID	Start	Stop	Protein Length (aa)	pI	Molecular weight	Localization
SmFtsH1	*SMEL4.1_04g023060.1.01*	82010033	82013843	708	6.29	76175.85	Chloroplast
SmFtsH4a	*SMEL4.1_12g017760.1.01*	75410244	75419444	708	9.00	76571.57	Chloroplast, Nucleus
SmFtsH4b	*SMEL4.1_00g001020.1.01*	4763311	4768569	683	7.79	73476.08	Chloroplast, Nucleus
SmFtsH6	*SMEL4.1_02g019910.1.01*	67601816	67604662	672	6.34	72895.61	Chloroplast
SmFtsH8	*SMEL4.1_07g019200.1.01*	101196708	101205295	783	6.02	83970.18	Chloroplast
SmFtsH9	*SMEL4.1_06g003380.1.01*	4829958	4843847	848	7.64	92834.33	Chloroplast, Nucleus
SmFtsH10a	*SMEL4.1_10g019040.1.01*	77705375	77712729	1060	7.05	118666.5	Nucleus
SmFtsH10b	*SMEL4.1_09g004580.1.01*	6695931	6707011	817	8.38	90420.19	Chloroplast, Nucleus
SmFtsH10c	*SMEL4.1_10g019030.1.01*	77684566	77701023	807	6.88	89010.19	Chloroplast, Nucleus
SmFtsH11	*SMEL4.1_03g018770.1.01*	82478763	82498029	814	5.96	89336.15	Chloroplast, Nucleus
SmFtsH12	*SMEL4.1_10g007120.1.01*	12613993	12634706	736	5.75	84376.04	Chloroplast, Nucleus
SmFtsHi1	*SMEL4.1_08g025960.1.01*	89918072	89926205	955	8.76	108116.6	Mitochondrion, Nucleus
SmFtsHi2a	*SMEL4.1_08g022650.1.01*	86211511	86223576	1195	9.53	137198.4	Mitochondrion, Nucleus
SmFtsHi2b	*SMEL4.1_05g012920.1.01*	57265803	57274725	743	6.85	83762.35	Mitochondrion, Nucleus
SmFtsHi3	*SMEL4.1_03g005230.1.01*	9379233	9387275	712	9.53	79346.27	Chloroplast, Nucleus
SmFtsHi4	*SMEL4.1_04g012340.1.01*	64559616	64572867	460	6.05	50870.34	Nucleus
SmFtsHi5	*SMEL4.1_02g003590.1.01*	35674907	35725329	1248	9.04	144065.1	Chloroplast, Nucleus

Table S4. Statistics of RNA-seq Data from Six Pepper Libraries.

Sample	Clean Reads	Q20(%)	Q30(%)	GC(%)	Mapped reads(%)	Uniq mapped(%)
T1	24,872,427	97.6	93.2	42.5	95.69	87.53
T2	29,377,017	97.8	93.9	42.7	95.92	87.35
T3	35,046,230	97.7	93.7	42.8	95.88	87.61
F1	36,009,410	97.6	93.4	42.8	95.67	86.33
F2	32,040,550	97.7	93.5	42.5	95.56	87.16
F3	40,134,886	97.7	93.5	42.9	95.94	87.55

Table S5. Relative Expression of 22 Selected Genes in Transcriptome Data.

Gene	T1	T2	T3	F1	F2	F3
Capana05g002549	1237.94	1792.26	1477.36	663.12	478.66	402.64
Capana10g000141	2430.74	2345.39	3033.43	809.11	725.34	588.44
Capana01g000647	603.49	884.73	1372	73.15	59.94	47.35
Capana03g000797	1198.64	1429.52	1856.99	605.76	512.44	468.88
Capana07g001245	5920.67	6806.31	6459.39	1964.96	1743.19	1710.1
Capana09g000473	661.1	893.11	1370.47	163.2	155.31	53.74
Capana07g002240	553.13	646.26	858.25	106.49	105.5	102.29
Capana03g003212	2554.05	2860.25	2788.41	691.01	648.48	586.87
Capana01g002398	2010.89	2273.17	2943.2	1059.45	1042.7	860.79
Capana08g002012	754.51	942.9	1334.37	147.71	125.32	121.46
Capana08g002008	1875.56	2546.32	3252.39	289.65	241.13	218.64

Capana04g000192	718.46	1012.39	1428.42	402.66	355	305.02
Capana02g001322	995.76	1280.05	1614.11	523.95	482.15	361
Capana07g002445	2517.16	2836.93	4114.5	1302.42	1201.48	884.41
Capana01g003910	4037.65	5064.63	5255.3	1477.39	1295.7	1125.28
Capana01g004493	2684.79	3410.75	3944.33	830.07	706.66	578.22
Capana06g000621	2533.61	3308.95	3572.43	1137.79	1007.12	893.3
Capana02g002133	2005.86	2487.68	2615.09	1465.9	1301.26	1047.23
Capana09g002353	5547.99	6407.96	7444.12	2612.61	2317.18	2186.65
Capana10g002492	1626.02	2071.59	2425.8	668.99	655.56	515.55
Capana04g000110	202.51	179.36	276.57	26.86	26.39	22.5
Capana11g000842	29.29	31.59	37.93	34.64	34.96	39.45

Table S6. All Primers Utilized in this Research.

Primer	Sequences（5′ to 3′）	Purpose
Ca04g000110-RT1F	GCTCTTGATGATGCAGCTCC	Expression pattern
Ca04g000110-RT1R	CAAGAACTCACTATACCTCC	analysis of *CaFtsH1*
Ca07g001803-RT1F	CCCTGAACCAACTATTGACGG	Expression pattern
Ca07g001803-RT1R	GATCTCTGTTCTTCCCTTGA	analysis of *CaFtsH8*
Ca-UBI-3F	TGTCCATCTGCTCTCTGTTG	Reference gene of
Ca-UBI-3R	CACCCCAAGCACAATAAGAC	RT-qPCR
CaFtsH1-C83-SL1F	gtcgactctagaggatccATGGCTAAATCTCTACTCTCTTCCAAT	Vector
CaFtsH1-C83-SL1R	actcatttttctaccggtaccGTTGAAATGTATAGCTCAGCCTTGCC	construction
CaFtsH8-C83-SL1F	gtcgactctagaggatccATGGCTACTTCATCAGTATGCATAGC	
CaFtsH8-C83-SL1R	actcatttttctaccggtaccGTTACGGCTGCTGGGGTAGGTACAGCAGC	Vector construction
CaFstH1-VIGS-1F	ctagatgaggagaagagcccATGGCTAAATCTCTACTCTCTTCCAAT	Vector construction
CaFstH1-VIGS-1R	tgtgctcgacgacaagacccGTTGGGGTTTGGTGCTTCAAC	
CaFstH8-VIGS-2F	ctagatgaggagaagagcccATGGCTACTTCATCAGTATGC	Vector construction
CaFstH8-VIGS-2R	tgtgctcgacgacaagacccCTTTTCCATTACCTAACAAAGC	
AD-FtsH1-1F	gccatggaggccagtgaattcATGGCTAAATCTCTACTCTCTTCCAAT	Vector construction
AD-FtsH1-1R	attcatctgcagctcgagctcTCATGAAATGTATAGCTCAGCCTTG	
BD-CaENGA-1F	atggccatggaggccgaattcATGGACCTCAGTCCAGCACTTC	Vector construction
BD-CaENGA-1R	ctagttatgcggccgctgcagTCATGTTGAAACTGCCAAACGT	
CaRT-1F	GGGTTACTCTAAAGACCCAAAG	RT-qPCR analysis of
CaRT-1R	CCAAGTGAGTTGCCAAGTTCTCC	*Capana05g002549*
CaRT-2F	GGCCACTCTGGTTTGCATCC	RT-qPCR analysis of
CaRT-2R	CTACCATAGCGCAACGCCCG	*Capana10g000141*
CaRT-3F	GAGCTACAGTTTGCCTCCTAA	RT-qPCR analysis of
CaRT-3R	CCTGTCACATTGTGCTGCAC	*Capana01g000647*

CaRT-4F	GAATGTGGATACCCTGGTGG	RT-qPCR analysis of
CaRT-4R	GGTCCCTTTCCAGTGACATTGTG	*Capana03g000797*
CaRT-5F	GGCAGCTTCAATGGAGGCCG	RT-qPCR analysis of
CaRT-5R	GTCCCATCCGTAGTCACCTGGG	*Capana07g001245*
CaRT-6F	CGTATCACCATGAGACGTACTGTC	RT-qPCR analysis of
CaRT-6R	GGATCGGCTGAGAGTCCAGCAGTG	*Capana09g000473*
CaRT-7F	ACGGATGGGACACTGCTGGT	RT-qPCR analysis of
CaRT-7R	CCATACTGGCTCCTTGAAGTCCAC	*Capana07g002240*
CaRT-8F	GGTGATTACGGATGGGACACTGC	RT-qPCR analysis of
CaRT-8R	CTGGTTCTTTGAAGTCCACC	*Capana03g003212*
CaRT-9F	GCTGTCATCGCAGAGGTTGTTC	RT-qPCR analysis of
CaRT-9R	CCTTCAATATTGCTGCCTGG	*Capana01g002398*
CaRT-10F	GTCCTGCTGTTAGAGGTGGTGGC	RT-qPCR analysis of
CaRT-10R	CTGCAGCCATTGCCCATCTGCC	*Capana08g002012*
CaRT-11F	CCTGGAGTAAGAGCTGGTGGC	RT-qPCR analysis of
CaRT-11R	CTGCAGTCATTGCCCATCTGCC	*Capana08g002008*
CaRT-12F	CCTCCTGCTAAAGCTGCCAAGCC	RT-qPCR analysis of
CaRT-12R	CCTCACTACAACTGGGTAGC	*Capana04g000192*
CaRT-13F	GAGGGAGAAGCAACAGATCAAG	RT-qPCR analysis of
CaRT-13R	CAGGTGTGGCAGTCCATCCG	*Capana02g001322*
CaRT-14F	GACAAACTCAAACCTTGGTGGC	RT-qPCR analysis of
CaRT-14R	CTGTGATGGGACTTGTTTGGC	*Capana07g002445*
CaRT-15F	CAGTGGCCTCAAACCCCAAGC	RT-qPCR analysis of
CaRT-15R	TCCAGCAAATAACATTAGGG	*Capana01g003910*
CaRT-16F	CGCGCTCCTCAGCCTTGCTGC	RT-qPCR analysis of
CaRT-16R	TGAACTGTGTATGCTCTTGC	*Capana01g004493*
CaRT-17F	TGGAAAGAGCCTTACTGGGC	RT-qPCR analysis of
CaRT-17R	AGTCCTCGGTCCGTCCCTTG	*Capana06g000621*
CaRT-18F	CGCTATGACCTTAAGACTGTCATC	RT-qPCR analysis of
CaRT-18R	GGTAGATACGGTAGCAGCATAG	*Capana02g002133*
CaRT-19F	CATCTGTTGTTGGATTGCCATC	RT-qPCR analysis of
CaRT-19R	CGGTGCTCATTCTCTCATCCAC	*Capana09g002353*
CaRT-20F	GCAGCTCAACAAATAGCCGA	RT-qPCR analysis of
CaRT-20R	CTAGCCCGGTAAGACCAAGTCCG	*Capana10g002492*
